# Validation of the Memorial Sloan Kettering Cancer Center nomogram to predict disease-specific survival in a Chinese gastric cancer population receiving postoperative chemoradiotherapy after an R0 resection

**DOI:** 10.18632/oncotarget.11665

**Published:** 2016-08-29

**Authors:** Meng-long Zhou, Lei Wang, Jia-zhou Wang, Wang Yang, Ran Hu, Gui-chao Li, Wei-qi Sheng, Zhen Zhang

**Affiliations:** ^1^ Department of Radiation Oncology, Fudan University Shanghai Cancer Center, Shanghai, 200032, PR China; ^2^ Department of Pathology, Fudan University Shanghai Cancer Center, Shanghai, 200032, PR China; ^3^ Department of Oncology, Shanghai Medical College, Fudan University, Shanghai, 200032, PR China

**Keywords:** gastric carcinoma, nomogram, postoperative chemoradiation, patient selection, prognosis

## Abstract

The widely validated Memorial Sloan Kettering Cancer Center (MSKCC) nomogram for gastric carcinoma (GC) was developed based on patients who received R0 resection only. The purpose of the current study was to assess the performance of this nomogram in Chinese patients who received postoperative chemoradiotherapy (CRT) after an R0 resection for GC. From 2006 to 2015, the clinical data of 150 eligible patients were retrospectively collected from the Fudan University Shanghai Cancer Center (FUSCC) and used for external validation. The nomogram was validated by means of the concordance index (CI) and a calibration plot. The CI for the nomogram was 0.657, which was lower than the CI of the nomogram for patients who received surgery alone (0.80). In the calibration plot, the gap between the observed and the predicted survival gradually increased as the predicted 5-year disease-specific survival (DSS) decreased. Thus the MSKCC nomogram for GC significantly underestimated the survival of patients in the FUSCC cohort, especially the survival of patients whose predicted 5-year DSS was less than 50%. The current study indicates the potential for the nomogram to be developed as an ideal tool to identify target patients for postoperative CRT.

## INTRODUCTION

Despite its decline in incidence over the past century, gastric carcinoma (GC) remains the second leading cause of cancer-related mortality worldwide and the most prevalent cancer in East Asia [1]. Surgical resection is considered the primary curative approach for this disease. However, even after radical resection, the loco-regional recurrence rate currently ranges from 24% to 54% [2], indicating that the effectiveness of surgery alone remains poor and unsatisfactory.

During the past two decades, combined modality therapy to prevent recurrence and improve survival in GC patients after curative resection has been widely investigated. Supported by the INT-0116 trial [3, 4], postoperative chemoradiotherapy (CRT) is now considered a standard treatment for patients with locally advanced GC who underwent an R0 resection without preoperative chemotherapy. However, the results of the ARTIST trial [5, 6] demonstrated that postoperative CRT does not provide a survival benefit to an unselected group of GC patients after R0 resection with D2 lymphadenectomy. The subgroup analyses of this trial showed that patients with positive lymph nodes or intestinal type GC may benefit from postoperative CRT, which indicates the importance of identifying target patients for postoperative CRT.

Nomograms estimate the survival probability of individual patients based on the patient, tumor, treatment, and pathology characteristics, and several nomograms have been developed for GC to date [7–10]. For example, the Memorial Sloan Kettering Cancer Center (MSKCC) nomogram for GC has been widely validated and proven to be robust [11–14]. It predicts the 5-year and 9-year disease-specific survival (DSS) after an R0 resection without any other therapy. Therefore, this nomogram may be an ideal tool for identifying patients who should undergo postoperative treatment. The results of the study conducted by Dikken et al. showed that this nomogram underestimated the DSS by approximately 20% for patients receiving postoperative CRT in western countries [15]. However, because the clinicopathologic characteristics and treatments of GC vary significantly between Asian and western countries, the performance of this nomogram for Chinese patients who have undergone surgery followed by postoperative CRT remains unknown. Therefore, the purpose of the current study was to assess the performance of the MSKCC nomogram for patients at a single high-volume center in China who received postoperative CRT after an R0 resection.

## RESULTS

### Patient selection

Between January 1, 2006 and June 30, 2015, 224 patients who underwent surgery at our center received postoperative CRT. However, 74 patients were excluded after a careful review by two independent reviewers (MLZ and WY), leaving 150 eligible patients for analysis. The reasons for exclusion were as follows: received preoperative chemotherapy (n=16), treatment discontinued due to severe toxicities or bad compliance (n=6), resection with residual disease (n=14), CRT given in a palliative setting (n=22), incomplete recordings (n=14), or loss to follow-up (n=2).

### Clinicopathologic characteristics

The clinicopathologic characteristics of the patients in the Fudan University Shanghai Cancer Center (FUSCC) cohort are summarized in Table 1. Most patients underwent a D2 lymphadenectomy (86.7%). The majority of patients had a tumor in the middle (34.7%) or distal stomach (42.0%). The disease of half (50.0%) of the patients were classified as diffuse-type GC. Patients with pathologic stage IIIC disease composed the largest proportion of the cohort (44.0%). The last follow-up date was March 31, 2016 and 60 patients (40.0%) had died of GC within a median follow-up period of 49 months.

**Table 1:** Patient characteristics of the FUSCC cohort (n=150)

Variables	Cases	(%)
Gender		
Male	105	70.0
Female	45	30.0
Age		
Mean (Range)	53.4	(25-77)
Median (IQR)	55	(47-61)
Primary site		
GEJ	21	14.0
Proximal	14	9.3
Middle	52	34.7
Distal	63	42.0
Lauren's classification		
Intestinal	44	29.3
Diffuse	75	50.0
Mixed	31	20.7
Invasion depth		
Mucosa	2	1.3
Submucosa	4	2.7
Muscularis propria	14	9.3
Subserosa	25	16.7
Serosal invasion	98	65.3
Adjacent organ invasion	7	4.7
N stage^*^		
N0	11	7.3
N1	20	13.3
N2	33	22.0
N3a	58	38.7
N3b	28	18.7
Tumor size (cm)		
Mean (Range)	4.3	(1.0-12.0)
Median (IQR)	4.0	(3.0-5.1)
Lymphadenectomy		
Less than D2	20	13.3
D2 or more	130	86.7
Positive LNs		
Mean (Range)	9.2	(0-32)
Median (IQR)	7	(4-14)
Negative LNs		
Mean (Range)	15.2	(0-52)
Median (IQR)	13	(9-20)
Total LNs		
Mean (Range)	24.4	(6-62)
Median (IQR)	23	(17-29)
TNM stage^*^		
IA	0	0
IB	1	0.7
IIA	8	5.3
IIB	17	11.3
IIIA	29	19.3
IIIB	29	19.3
IIIC	66	44.0

Abbreviation: IQR = interquartile ratio; GEJ = gastroesophageal junction; LNs = lymph nodes.

* according to AJCC 7^th^ edition

### Evaluation of the MSKCC nomogram

**Figure 1:**
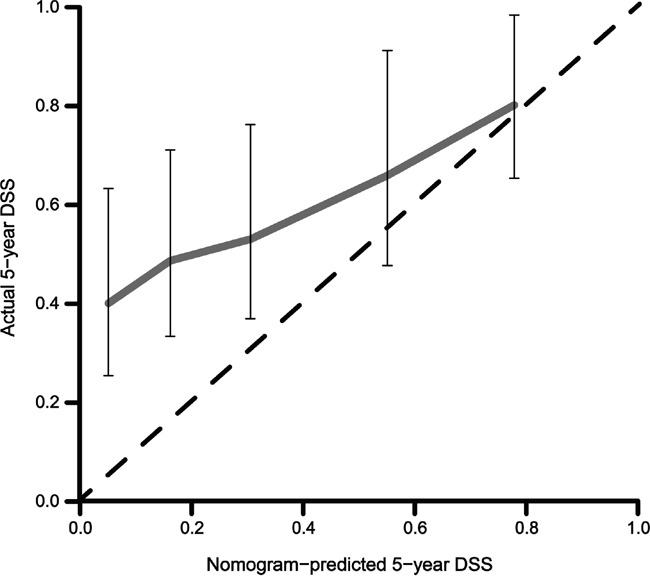
Calibration plot of the nomogram validated in patients who received postoperative chemoradiotherapy after an R0 resection (n=150) DSS: disease-specific survival.

The performance of the MSKCC nomogram in the current patient cohort was evaluated using two methods. First, discrimination between individual patients was assessed with the concordance index (CI), which was 0.657 for this model (95% CI: 0.651-0.662). Second, a calibration plot compared the nomogram-predicted probability of the DSS with the observed rate of DSS at 5 years (Figure 1). The performance of the ideal nomogram is indicated by the dotted line, whereas the solid line represents the performance of the MSKCC nomogram in predicting the DSS probabilities of patients in the FUSCC cohort who received CRT after R0 resection. The calibration plot showed that the deviation from the actual probability inversely correlated with the nomogram-predicted probability. Therefore, the nomogram significantly underestimated the 5-year DSS probabilities in the current patient cohort, especially for patients with low nomogram-predicted probabilities.

**Figure 2:**
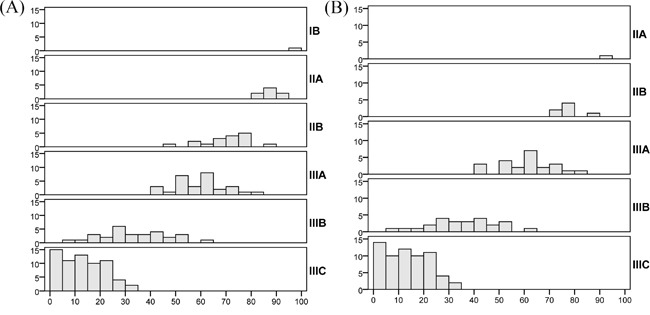
Histograms of the MSKCC nomogram-predicted probabilities within each AJCC stage The x-axis represents the nomogram-calculated probability of the 5-year DSS, and the y-axis represents the number of patients within each interval of the 5-year DSS. The bold markers on the right side of each histogram represent the AJCC stage of gastric cancer according to the seventh edition. **A.** All patients in the FUSCC cohort (n=150); **B.** Patients who underwent R0 and D2 resection with stage II-III disease and positive lymph nodes (n=119).

We then compared the predictive ability of the MSKCC nomogram with that of the seventh AJCC stage risk grouping. Specifically, the CI for the AJCC stage was 0.642 (95% CI: 0.637-0.647). Although the p value showed that the MSKCC nomogram was more discriminative than the seventh AJCC TNM classification (concordance index, nomogram vs. AJCC stage: 0.657 vs. 0.642; p=0.000), the actual difference was not readily apparent. To illustrate the discrepancies between the two predicting methods, Figure 2A shows a histogram of the nomogram-predicted survival probabilities for each AJCC stage, which suggests heterogeneity within several AJCC stages, particularly stages IIB, IIIA, and IIIB. Even for patients with stage II-III disease and positive lymph nodes, each AJCC stage overlaps with the neighboring AJCC stages (Figure 2B).

## DISCUSSION

In the current study, the performance of the MSKCC nomogram was evaluated for Chinese GC patients who received postoperative CRT after an R0 resection. The CI for this model (0.657), which indicates the discriminative ability, was lower than that of the nomogram for patients who did not receive adjuvant therapy (0.80) [17], but similar to that in the MSKCC/NKI cohort (0.64) [15]. Therefore, the discriminative ability remained moderately high in this eastern population. The survival was underestimated by the MSKCC nomogram in the FUSCC cohort, as anticipated. The difference in the performance between the eastern and western cohorts is visually illustrated by the calibration plots at five years. Specifically, the calibration plot of the MSKCC/NKI cohort consistently showed an approximately 20% higher survival rate than the nomogram-predicted survival for patients receiving postoperative CRT. However, in the calibration plot of the FUSCC cohort, the gap between the observed and the predicted survival gradually widened as the predicted survival decreased, suggesting that patients with a low predicted 5-year DSS may benefit more from postoperative CRT.

The patient characteristics were compared between the FUSCC and MSKCC/NKI cohorts (Table 2), and this comparison revealed several significant differences between the two cohorts. Specifically, the disease stages were more advanced (p=0.014) and more diffuse-type GCs were diagnosed (50.0% vs. 38.9%, p=0.105) in the FUSCC cohort. Moreover, patients with stage IIIC disease composed 44.0% and 26.6% of the FUSCC and MSKCC/NKI cohorts, respectively. In addition, more positive lymph nodes were retrieved from R0 resection in the FUSCC cohort (median: 7 vs. 4; inter quartile range: 4-14 vs. 2-10). This difference may be attributed to the high rate of D2 lymphadenectomy in the FUSCC cohort (86.7%), and this rate was not available for the MSKCC/NKI cohort. Although patients in both cohorts received postoperative CRT, the chemotherapy (ChT) regimens, courses and concurrent ChT differed. In the MSKCC/NKI cohort, the majority of patients (n=46) received 5-FU with leucovorin (LV) according to the INT-0116 protocol, whereas most patients in the FUSCC cohort (n=66) received epirubicin (EPI) and oxaliplatin (OXA) with 5-FU according to the regimen specified in the MAGIC trial [18], and cisplatin (DDP) was replaced by OXA. Moreover, S-1, a widely accepted cytotoxic agent in Asia, was only used in the FUSCC cohort. In addition, the radiotherapy (RT) target differed. In the MSKCC/NKI cohort, the clinical target volume (CTV) routinely included the remnant stomach, which had not been targeted in the FUSCC cohort since 2008.

**Table 2:** Comparison of patients' clinicopathologic characteristics in the FUSCC and MSKCC/NKI cohorts

Variables	FUSCC (n=150)		MSKCC/NKI (n=139)		p-value
	Cases	%	Cases	%	
Gender					0.899
Male	105	70.0	96	69.1	
Female	45	30.0	43	30.9	
Age					
Median (IQR)	55	47-61	61	51-68	
Primary site					0.818
GEJ	21	14.0	16	11.5	
Proximal	14	9.3	17	12.2	
Middle	52	34.7	47	33.8	
Distal	63	42.0	59	42.5	
Lauren's classification					0.105
Intestinal	44	29.3	56	40.3	
Diffuse	75	50.0	54	38.9	
Mixed	31	20.7	29	20.9	
Invasion depth					0.218
Mucosa	2	1.3	1	0.7	
Submucosa	4	2.7	7	5.0	
Muscularis propria	14	9.3	13	9.4	
Subserosa	25	16.7	34	24.5	
Suspected serosal invasion	-	-	21	15.1	
Serosal invasion	98	65.3	58	41.7	
Adjacent organ invasion	7	4.7	5	3.6	
Tumor size					
Median (IQR)	4.0	(3-5.1)	5	(2.9-6.5)	
Positive LNs					
Median (IQR)	7	(4-14)	4	(2-10)	
Negative LNs					
Median (IQR)	13	(9-20)	11	(4-20)	
TNM stage^*^					0.014
IB	1	0.7	3	2.2	
IIA	8	5.3	7	5.0	
IIB	17	11.3	20	14.4	
IIIA	29	19.3	39	28.1	
IIIB	29	19.3	33	23.7	
IIIC	66	44.0	37	26.6	

Abbreviation: IQR = interquartile ratio; GEJ = gastroesophageal junction; LNs = lymph nodes.

* according to AJCC 7^th^ edition

The INT-0116 trial [4] demonstrated the efficacy of postoperative CRT compared with surgery alone for the treatment of advanced resectable GC. However, this trial was criticized due to the low rate (10%) of D2 lymphadenectomy. Therefore, it cannot be directly referred to in Asian countries, where postoperative ChT and D2 lymphadenectomy are widely performed. Moreover, the ARTIST trial [6], which was designed to compare postoperative CRT with ChT in advanced GC after D2 and R0 resection, failed to prove the efficacy of postoperative CRT. However, patients with stage Ib and II disease composed nearly 60% of the cohort in both groups, which may dilute the observed survival benefit of CRT. Given the default limitations in its trial design, the role of postoperative CRT after D2 and R0 dissection remains undefined. Furthermore, subgroup analyses of the ARTIST trial [5] demonstrated the potential benefit provided by the addition of RT to postoperative ChT in patients with node-positive disease and intestinal-type GC. This finding implies that selecting patients who would benefit from postoperative CRT will be paramount.

Target patients for postoperative CRT may have one or more of the following characteristics: non-optimized surgery, advanced disease stage, intestinal-type GC, and lymph node metastasis [19]. Surgery is the key component of multimodal treatment for GC, and postoperative CRT may significantly reduce the risk of loco-regional recurrence (LRR) for patients whose extent of lymphadenectomy is less than D2. Disease stage is another crucial factor for patient selection, even among patients who underwent D2 dissection. In a multivariate analysis by stage and treatment arm in the ARTIST trial, postoperative CRT resulted in significantly prolonged disease-free survival (DFS) (p<0.0471). A retrospective study conducted by Jin et al. [20] showed that CRT improves both the overall survival (OS) (p=0.041) and DFS (p=0.033) compared with postoperative ChT in patients with stage IIIC disease. Additionally, the Lauren classification and lymph node status have also been identified as crucial factors. Specifically, patients with intestinal-type GC are more likely to benefit from CRT than those with diffuse-type disease according to subgroup analyses of the INT-0116 and ARTIST trials. Several studies [21–23] have demonstrated that a diffuse adenocarcinoma is prone to distant metastasis, which cannot be effectively controlled by postoperative CRT. A retrospective study [24] using a propensity score matching method also demonstrated that positive lymph nodes are an indicator of potential benefit from postoperative CRT (p<0.001).

The MSKCC nomogram for GC has been widely validated and manifests a high predictive accuracy. Risk stratification is one function of this nomogram, which may help to select patients for postoperative therapy. Based on the results of the ARTIST trial, the subsequent ARTIST-2 trial [25] enrolls patients after D2 and R0 resection with pathologic stage II or III disease and positive lymph nodes. However, patients who meet this eligibility criteria have different nomogram-predicted 5-year DSS rates (Figure 2B). Specifically, most patients with stage IIA, IIB, or IIIA disease have a 5-year DSS of more than 50%, and the gap between the actual and predicted 5-year DSS is not as large as that of patients who have a 5-year DSS of less than 50%. This finding, to some extent, indicates that selecting patients only based on disease stage and lymph node status may be inefficient. Nevertheless, the ARTIST-2 trial may answer this question in the future. In contrast, a nomogram would increase the precision of patient selection because it incorporates multiple aforementioned clinicpathologic factors.

In addition to all clinical and pathologic factors, gene-based classifications and features should also be taken into account. For example, the ARTIST trial considered differences in the HER-2, MET, MLH1, and CDH1 gene statuses. Based on the underlying molecular biology of the tumor, the Cancer Genome Atlas (TCGA) project and the Asian Cancer Research Group (ACRG) have both proposed a four-subtype classification for GC [26, 27]. These studies have expanded our understanding of the characteristics of GC at the molecular level. The tendency for lymph node involvement, the development of a distant metastasis, and the radiosensitivity of tumor cells are all determined or influenced by genes that have not yet been wholly identified. The precision for selecting patients may also be improved by a nomogram that incorporates not only the clinical and pathologic factors but also the status of critical genes.

In the current study, the performance of the MSKCC nomogram for GC was validated in a cohort of 150 GC patients who received postoperative CRT after an R0 resection. The discriminative ability was moderately high in this Asian population, and the survival benefit provided by postoperative CRT inversely correlated with the nomogram-predicted DSS. This finding implied the potential of developing this nomogram as an ideal tool to select patients who may benefit from postoperative CRT. The number of patients in the FUSCC cohort was sufficient to assess the performance of the MSKCC nomogram, but it was too low to create a new nomogram specifically for patients who received postoperative CRT. At present, we are attempting to collaborate with other large centers collecting data on patients with GC who are treated with multimodal therapy to create such a nomogram.

However, this study was subject to some limitations. First, in practice, it is difficult to distinguish tumors of the gastroesophageal junction (GEJ) from tumors of the proximal or upper one-third (P/U) of the stomach. Second, none of the patients in our cohort were classified to suffer from suspected serosal invasion because its definition is unclear. Third, 20% of patients had a record of treatment after recurrence in the FUSCC cohort and the effect of therapy after recurrence on survival was difficult to evaluate.

In conclusion, we externally validated the ability of the MSKCC nomogram to predict the probability of 5-year DSS after R0 resection and postoperative CRT for GC in a single Chinese cohort database. Specifically, the nomogram significantly underestimates the 5-year DSS, especially for patients whose predicted 5-year DSS is less than 50%. Postoperative CRT after R0 resection may be indicated for these patients because they may derive the most benefit from this treatment.

## PATIENTS AND METHODS

### Ethics statement

This retrospective study was approved by the research ethics committee of the FUSCC. Written informed consent was obtained from all patients prior to this study.

### Patients

Between January 1, 2006 and June 30, 2015, more than 5,000 consecutive patients who underwent a resection for an adenocarcinoma of the stomach or GEJ at the FUSCC were retrospectively reviewed. Of these patients, 224 received postoperative CRT. A R0 resection was defined as complete resection without a microscopic residual tumor. Patients were excluded from the study if the final pathology report revealed a positive surgical margin or metastatic disease. Patients who received preoperative treatment and patients who did not undergo an R0 resection were excluded.

### Treatments

**Figure 3:**
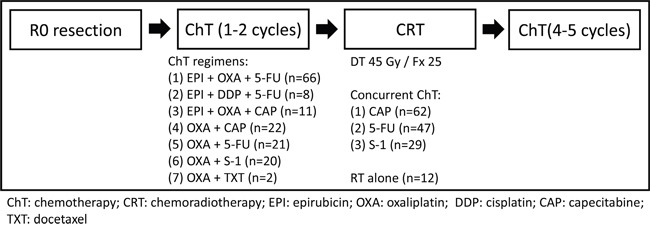
Flowchart of the treatment and the chemotherapy regimens

All patients underwent a total or subtotal gastrectomy, usually with a D2 lymphadenectomy, according to the Japanese Research Society for Gastric Cancer (JRSGC) guidelines. A routine splenectomy or pancreatic tail resection was not performed. Postoperatively, all patients received one or two courses of ChT, followed by CRT (45 Gy of radiation at 1.8 Gy per day, 5 days per week, for 5 weeks with concurrent ChT) and four to five additional subsequent courses of ChT (Figure 3).

RT was targeted to the tumor bed, anastomosis site, and regional lymph nodes. The planning target volume (PTV) margins were added by individually considering uncertainties. The remnant stomach was not routinely included within the radiation field since the year 2008, based on the results of a study from Korea [16].

The majority of patients (n=66) received EPI and OXA with 5-FU. The other patients received EPI and DDP with 5-FU (n=8), EPI and OXA with capecitabine (n=11), OXA with 5-FU (n=21), S-1 with OXA (n=20), capecitabine with OXA (n=22), and docetaxel with OXA (n=2). Fluorouracil-based ChT was concurrently used with RT, which included a continuous intravenous infusion of 225 mg/m^2^ of 5-FU for 120 hours each week (n=47), 625 mg/m^2^ capecitabine twice daily during RT (n=62) or 40 mg/m^2^ S-1 b.i.d. daily during RT (n=29). The remaining patients (n=12) received RT without concurrent ChT in consideration of the toxicities and tolerance of treatment.

### Follow-up

The regular follow-up program started after patients were discharged from the hospital. Patients were followed-up every 3–6 months during the first 2 years, every 6 months until the fifth postoperative year, and once annually thereafter. The follow-up evaluation consisted of a physical examination, radiological studies, an endoscopic examination, and a laboratory examination.

### Clinicopathologic variables

The patients' data were collected and included the following prognostic variables: sex, age at diagnosis, the primary site of the tumor (GEJ, proximal or upper third of the stomach, body or middle third of the stomach and antrum or pyloric), Lauren's histologic type (diffuse, intestinal, or mixed), tumor size, the number of resected positive lymph nodes, the number of resected negative lymph nodes, and the depth of tumor invasion (mucosa, submucosa, propria muscularis, subserosa, serosal invasion, and adjacent organ involvement). Patients were excluded due to lack of information or death from causes unrelated to GC.

### Statistical analysis

The DSS was calculated from the day of surgery until the day of death due to GC (event) or other causes until the day of the last follow-up (censored). For each patient, the nomogram-predicted 5-year DSS probability was computed. Nomogram validation consisted of two steps. First, discrimination was quantified with the CI, which is similar to the area under the receiver operating characteristic curve but is appropriate for censored data and ranges from 0.5 (no discrimination) to 1.0 (perfect discrimination). Given a randomly selected pair of patients, the CI is the probability that the patient who dies first had the worst predicted outcome according to the nomogram. Second, calibration plots were assessed by grouping patients with respect to their nomogram-predicted probabilities and then comparing the mean of the group with the observed Kaplan-Meier estimate of the DSS. A pairwise t-test was used to test differences in the CI between the TNM stage and nomogram. All analyses were performed using the R statistical software package (version 3.2.5).
